# Draft Genome Sequence of *Deinococcus* sp. Strain RL Isolated from Sediments of a Hot Water Spring

**DOI:** 10.1128/genomeA.00703-14

**Published:** 2014-07-17

**Authors:** Nitish Kumar Mahato, Charu Tripathi, Helianthous Verma, Neha Singh, Rup Lal

**Affiliations:** Department of Zoology, University of Delhi, Delhi, India

## Abstract

*Deinococcus* sp. strain RL, a moderately thermophilic bacterium, was isolated from sediments of a hot water spring in Manikaran, India. Here, we report the draft genome (2.79 Mbp) of this strain, which contains 62 contigs and 2,614 coding DNA sequences, with an average G+C content of 69.4%.

## GENOME ANNOUNCEMENT

Hot water springs located atop the Himalayan ranges in Manikaran, India, are known for harboring unique microbial diversity (1, 2). We have been exploring the microbial diversity of these hot water springs by using culture-dependent and culture-independent approaches. Earlier, we reported the genome sequence of *Thermus* sp. strain RL, isolated from water samples of this hot water spring (3). In continuation of our efforts, we isolated *Deinococcus* sp. strain RL from sediments of the same hot water spring in 2013 and sequenced its genome to form the basis for carrying out comparative genome analysis with other closely related radioresistant members of this genus (4, 5).

The genome of *Deinococcus* sp. strain RL was sequenced using the Illumina Genome Analyzer platform. Paired-end reads of 500 bp (*n* = 12,988,682) and 2 Kbp (*n* = 13,994,204) were assembled using *de novo* assembler ABySS (version 1.3.5) (6) at a *k*-mer of 57, resulting in 62 contigs (>500 bp with *N*_50_, 87,506 bp). The total length of the genome was estimated to be 2,792,068 bp, with an average G+C% of 69.4 and 2,614 coding DNA sequences. The assembled contigs were aligned on the paired-end raw reads using BWA (version 0.7.9a) (7) and validated using Tablet (version 1.14.04.10) (8). Annotation of the assembled genome was performed using the RAST server (version 4.0) (9) and NCBI Prokaryotic Genomes Annotation Pipeline (PGAP) (see http://www.ncbi.nlm.nih.gov/genomes/static/Pipeline.html).

The draft genome sequence has a coding density of 89.59%, featuring 344 subsystems and 18 pseudogenes. Three rRNA operons (5S-16S-23S) were predicted using RNAmmer (version 1.2) (10). Forty-nine aminoacyl-tRNA synthetase genes and one transfer-messenger RNA (tmRNA) gene were also predicted using ARAGORN (11). Further, *Deinococcus murrayi* DSM 11303 is found to be its closest neighbor using BLAST (12). The average nucleotide identity (13) between these two strains is 97.77%. Thirteen insertion sequence (IS) elements showing significant hits with IS elements of *Deinococcus geothermalis* were revealed using ISfinder. By using AMPHORA (14), we found 31 bacterial marker genes in the draft genome. Additionally, 14 confirmed clustered regularly interspaced short palindromic repeat (CRISPR) arrays were also identified in the draft genome.

The RAST subsystem analysis showed the presence of a minimum number of genes that are required to complete the pathway of carotenoid biosynthesis (15). Genes involved in base excision repair, nucleotide excision repair, mismatch repair, and homologous recombination were also identified. Further work to describe the metabolic profile of strain RL and comparative genomic analysis with its closest radioresistant neighbors is being carried out in order to depict genome plasticity at high temperature environments.

### Nucleotide sequence accession numbers.

This whole-genome shotgun project has been deposited at DDBJ/EMBL/GenBank under the accession no. JMQF00000000. The version described in this paper is version JMQF01000000.
